# Compositional and Functional Comparisons of the Microbiota in the Colostrum and Mature Milk of Dairy Goats

**DOI:** 10.3390/ani10111955

**Published:** 2020-10-23

**Authors:** Zhannur Niyazbekova, Xiao-Ting Yao, Ming-Jie Liu, Nomin Bold, Juan-Zhen Tong, Jian-Jun Chang, Ying Wen, Li Li, Yong Wang, De-Kun Chen, Wen-Tao Ma

**Affiliations:** 1 College of Veterinary Medicine, Northwest Agriculture and Forestry University, Yangling District, Xianyang 712100, China; zhannur_niyazbekova@nwafu.edu.cn (Z.N.); yaoxiaoting@nwafu.edu.cn (X.-T.Y.); mingjie.liu@nwsuaf.edu.cn (M.-J.L.); tongjuanzhen0819@nwsuaf.edu.cn (J.-Z.T.); 2 Biomass Energy Center for Arid and Semi-Arid Lands, State Key Laboratory of Crop Stress Biology for Arid Areas, College of Life Sciences, Northwest Agriculture and Forestry University, Yangling 712100, China; nomin@nwafu.edu.cn; 3 State Key Laboratory of Plateau Ecology and Agriculture, Qinghai University, Xining 810016, China; changjianjun2020@163.com (J.-J.C.); wenying547738878@163.com (Y.W.); llqhdx@163.com (L.L.); wangyong3572742491@163.com (Y.W.); 4 College of Agriculture and Animal Husbandry, Qinghai University, Xining 810016, China

**Keywords:** goat milk, colostrum, mature milk, milk microbiota, microbial diversity, high-throughput sequencing

## Abstract

**Simple Summary:**

Our findings revealed significant influences of lactation stage on goat milk microbial and functional composition both in colostrum and mature milk. Microbial communities which were found in colostrum and mature milk could potentially establish goat kids’ gut microbiota and have an influence on the development of microbial components that provide potential health promotion effects in goats.

**Abstract:**

Goat milk is essential for the initial development of kids by providing a great source of commensal bacteria. In this study, we analyzed the microbiota of the milk of 30 healthy Saanen dairy goats. The 30 samples comprised 15 colostrum and 15 mature milk samples, collected from three different farms of Shaanxi Province. Colostrum samples were collected daily for five days post-delivery and mature milk was collected on the 7th, 10th, 20th, 30th, and 40th days. The result showed that microbial alpha diversity was higher in the mature milk compared with that in the colostrum. Linear discriminant analysis effect size (LEfSe) was performed to detect differentially abundant taxa in colostrum and goat milk. According to taxonomy results, *Proteobacteria*, *Firmicutes*, *Actinobacteria*, and *Bacteroidetes* were the predominant bacteria phyla in both colostrum and mature milk. In addition, lactation stage noticeably influenced the composition of milk microbiota. Specifically, *Novosphingobium*, *Brachybacterium*, *Psychrobacter*, *Lactobacillus*, *Yersinia*, *Roseateles*, *Rothia*, *Sanguibacter*, *Cloacibacterium*, *Variovorax*, *Sphingobacterium*, and *Coxiella* were enriched in the colostrum, while *Georgenia*, *Peptostreptococcus*, *Bacteroidales*, *Yaniella*, *Planomicrobium*, *Cloacibacterium*, *Azospirillum*, *Turicibacter*, *Cupriavidus*, *Herbaspirillum*, *Rhodobacteraceae*, and *Aeromonadales* were the dominant genera in the mature milk. The enriched metabolic functions of the goat milk microbiota were predicted by PICRUSt and classified by KEGG pathway. Moreover, the abundances of environmental information processing, cellular processes pathway, genetic information processing pathway, organismal systems pathway, and metabolism pathway were significantly different between microbiota of colostrum and mature milk. Altogether, our study disclosed the significant difference between the microbial communities of colostrum and mature milk and provided grounds for further research in dairy microbiology.

## 1. Introduction

Goat milk production accounts for about 2.1% of global milk production. About 95% of the world’s goat population is located in Asia, Africa, and Latin America. Among them, Asia accounts for approximately 60% of the total goat population [1]. Goat milk production in China rose from 54,000 tons in 1969 to 223,134 tons by 2018 at a remarkable yearly average rate of 3.31% (https://knoema.com/data/china+agriculture-indicators-production+goats+milk) [2]. In China, Shaanxi Province possesses the largest storage facility for goat milk.

Goat milk contains relatively less lactose and fat but is rich in calcium, antimicrobial factors, antioxidants, and other functional components that are essential for the health of human beings [3]. Similar to human breast milk, goat milk contains a high level of antimicrobial enzymes, such as lysozyme, that boost the immunity of infants against numerous infections [4]. In addition, goat milk shares several key features with human breast milk and has been regarded as the most important replacement for human breast milk [5,6].

Colostrum is a nutrient-rich milk that, in particular, contains a large amount of immunoglobulin, lactoferrin, and a variety of other growth factors that are produced for a few days post-parturition. It provides necessary protection to newborns through passive immunity against pathogens. The colostrum ingestion by newborn ruminants is the only source of immunoglobulins (Ig) during the first month of life and is further maintained throughout their whole lifespan [7]. Moreover, not only metabolic and immunological alterations but also hormonal and nutritional changes occur over the transition period, which influence the incidence of infectious and metabolic diseases [8,9]. Lactation stages are mostly gradual but sometimes sudden changes may occur in composition and properties [10]. Colostrum is low in fat but high in protein and plays a crucial role in boosting the immune-protective components [11]. However, mature milk is characterized by a lower percentage of proteins and minerals and more richness in carbohydrates and lipids compared with colostrum [12]. By developing intestinal immune balance, the infants adapt to the extra-uterine environment. In order to complete the development of the intestinal immune system, an optimum amount of essential bacteria is very crucial in defining the fate of the infant health system [13]. Antimicrobial factors provide passive immunity and protection against infections. They nullify the pathogen agents directly or indirectly by stimulating the growth of a healthy intestinal microbiota [14]. Probiotic bacteria such as *Lactobacillus*, *Lactococcus*, and *Bifidobacterium* isolated from mammals have been used in the prevention and treatment of mastitis with similar or better efficacy than traditional antibiotic treatment [15].

The main goal of this research is to examine the microbial diversity and community in goat colostrum and mature milk, which were sampled at their different lactation stages from goat milk collected from different areas of Shaanxi Province. The findings of this study provide insightful knowledge about the generation of immune-specific microbiota in the infant gut. The development of gut microbiota is indispensably important for the normal health of infant goats.

## 2. Materials and Methods

### 2.1. Sample Collection

The study was approved by the Institutional Animal Care and Use Committee of the Northwest Agriculture and Forestry University under permit number 2019NWAFU1122, Yangling, China on 24 November 2019. The study was conducted and implemented in accordance with the Guide for the Care and Use of Laboratory Animals of the Ministry of Agriculture, China under proclamation No.5.

In this study, a total of 30 samples from 30 randomly selected goats from three local farms (farms A, B, and C) in Shaanxi Province of China (Table 1) were collected during the spring of 2019. All animals in this experiment were healthy and were provided with normal feeding materials. The samples, including colostrum and mature milk, were collected from each animal at 8 a.m., before their milking. The colostrum samples (*n* = 15) were collected on the first five days after the delivery and mature milk samples (*n* = 15) were collected on the 10th, 20th, 30th, and 40th days.

Before conducting the manual milk sample collection, the udder surface of each goat was washed and sterilized with 70% ethyl alcohol and then the sample was collected into a sterile plastic tube after discarding the first drop of milk. Each sample was numbered and refrigerated immediately at 4 °C and was transported to the laboratory inside an icebox.

### 2.2. DNA Extraction

The microbial community in 1 mL of each milk sample was centrifuged at 12,000 rpm for 5 min and the pellets were suspended three times in 200 µL 0.8% NaCl solution and bacterial genomic DNA was isolated by the cetyltrimethylammonium bromide (CTAB) method [16]. The pellet was resuspended in 500 µL CTAB buffer (20 g CTAB/L, 1.4 M NaCl, 0.1 M Tris/HCl, 20 mM EDTA) and this mixture was incubated for 30 min at 65 °C. Bacteria were pelleted by centrifugation at 9000 rpm for 10 min and pellets were resuspended in CTAB precipitation solution (5 g CTAB/L, 0.04 M NaCl). Then samples were incubated for 60 min at room temperature. After incubation, samples were centrifuged at 9000 rpm for 5 min, the supernatant was removed, the chloroform was used for DNA isolation, and that was frozen at −80 °C till the next experiment. The DNA bands were identified in 0.8% (w/v) agarose gel and each DNA concentration was measured by a NanoDrop 2000 spectrophotometer (Thermo Fisher Scientific, Waltham, MA, USA).

### 2.3. Amplification of V3 and V4 Regions of 16S rRNA Gene

The V3 and V4 regions in 16S rRNA genes were determined with the specific primers 338F: 5′- ACTCCTACGGGAGGCAGCA -3′; 806R: 5′- GGACTACHVGGGTWTCTAAT -3′ with a nucleotide barcode, respectively. Each PCR reaction amplified about 10 ng of template DNA along with 0.2 µL of each primer in 15 µL of Phusion High-Fidelity PCR Master Mix (New England Biolabs, Ipswich, Massachusetts, United States). PCR thermal cycling was done at 98 °C as an initial denaturation for 1 min followed by 30 cycles of 98 °C for 10 s for denaturation, 50 °C for annealing for 30 s, and 72 °C for elongation for 30 s in each cycle and amplification was terminated by a 5 min final extension at 72 °C. The amplified PCR products were joined with Synergy Brands (SYBR) green dye and specific products (between 200 bp and 450 bp) were determined in 2% agarose gel and purified from the gel by a Qiagen Gel Extraction Kit (Qiagen, Hilden, Nordrhein-Westfalen, Germany). 

### 2.4. Library Preparation and Sequencing

Sequencing libraries were prepared by using a TruSeq DNA PCR-Free Sample Preparation Kit (Illumina, San Diego, CA, USA) with unique indices according to the manufacturer’s instructions. The 5′ ends of the amplificons were removed by using the Fast DNA End Repair Kit (Thermo Scientific, Waltham, MA, USA), and overhanging adenine residues were added at the 3′ blunt end and then phosphorylated at the 3′ end which prevented the DNA fragment from self-joining. The termination sequence contained library-specific tags (i.e., index sequence) at the 5′ end so that DNA molecules could be modified on the flow cell. PCR was performed on the modified DNA template constructed with the termination sequence to amplify the sequencing library template, and then AMPure XP Beads (BECKMAN Coulter, Indianapolis, IN, USA) were used to purify the enrichment products of the library. The final fragment of the library selection and purification was conducted by gel electrophoresis in 2% gel. The quality of the library assessment was performed on a Qubit 2.0 Fluorometer (Thermo Scientific) and Agilent Bioanalyzer 2100 (Agilent Technologies, Santa Clara, CA, USA) system according to the manufacturer’s instructions. In the end, the sequencing of the library was completed and 480 bp paired-end sequences were generated on an Illumina MiSeq-PE250 platform. 

### 2.5. Bioinformatical and Statistical Analysis

In this study, an Illumina MiSeq-PE250 platform was used to conduct paired-end sequencing on the DNA fragments and original sequencing data were converted into FASTQ format [17]. The sequence data from different samples were separated based on the barcode sequences. FLASH software (V1.2.7, http://ccb.jhu.edu/software/FLASH/) was used to assemble reads that were barcode and primer free to obtain the raw tags. Raw tag quality filtering was performed to obtain high-quality tags by following the Quantitative Insights Into Microbial Ecology (QIIME) guidelines [18,19]. In order to detect and remove chimera sequences, the tags were compared to the SILVA database by the UCHIME algorithm [20] and removed [21]. Then effective tags were obtained and clustered into operational taxonomic units (OTUs) by UPARSE software [22]. For instance, ≥97% similar sequences were designated to the same operational taxonomic units (OTUs) [23]. The representative sequence for each OTU was evaluated for further analysis and each representative sequence was applied for taxonomic annotation based on the Mothur algorithm in the SILVA database [24]. Multiple sequence comparison by log-expectation (MUSCLE) software was used for the study of phylogenetic relationships of different OTUs and differences in dominant species in different samples [25].

With the corresponding standard sequence, the community abundance information of OTUs was assimilated, in which subsequent analysis was confirmed for alpha and beta diversity. The diversity of species and its complexity were analyzed through alpha diversity with Shannon and Simpson indices. Beta diversity estimation was performed by Bray–Curtis distance matrices and visualized by principal coordinate analysis (PCoA) [26,27]. The indices mentioned above were computed by QIIME (version 1.7.0) and represented by R software (version 3.6.1, TUNA Team, Tsinghua University, Beijing, China).

Variations of abundant taxa and functions between the groups were characterized by the linear discriminant analysis effect size (LEfSe) in accordance with taxonomic and functional profiles of genes (http://huttenhower.sph.harvard.edu/galaxy/) [28].

Different environments could affect the milk microbiota composition and diversity [29]. Therefore, we analyzed all the samples independently in order to avoid variation because of the environment. By doing this, we can know the microbial load in each sample of mature milk and colostrum separately.

In addition, the phyloseq package [30] was used to plot data and perform statistical analysis in R. Metagenome function was predicted by Phylogenetic Investigation of Communities by Reconstruction of Unobserved States (PICRUSt) software [31]. Metabolic pathways were aligned and translated from the gene catalog against the proteins (or domains) in the KEGG databases (http://www.genome.jp/kegg/). In order to compare significant differences in the microbiota between colostrum and mature milk of goats, the Wilcoxon rank-sum test with phyloseq was run in R software.

## 3. Results

### 3.1. Differences of the Microbial Diversity and Compositions in Colostrum and Mature Milk from Different Farms

**Figure 1:**
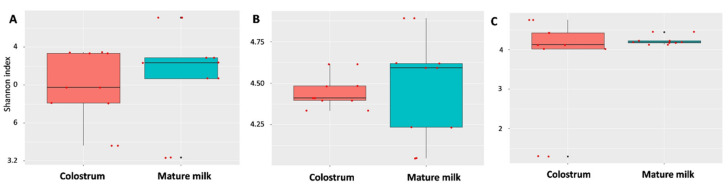
Alpha diversity measured (within sample diversity) by Shannon index, statistical (*p*) value generated by Wilcoxon test. The richness of the alpha diversity bacterial community in mature milk was higher than that in colostrum, but these differences were not statistically significant for farm A (**A**) (*p* = 0.84), B (**B**) (*p* = 0.84), or C (**C**) (*p* = 0.56). The horizontal bars within boxes represent median values, the tops and bottoms of boxes represent 75th and 25th quartile values, respectively.

We obtained 1,010,969 bacterial sequences from the 30 different colostrum and mature milk samples. Among them, the median number of reads in colostrum was 498,864, and that in the mature milk was 512,105. All these reads were classified into 15,430 OTUs, which were used for downstream analyses. The indices of bacterial alpha diversity in the colostrum and mature milk of dairy goats are listed in Figure 1. There was no consequential difference in the Shannon index (Figure 1A–C) and Simpson index (Figure S1) between the colostrum and mature milk from different farms. The Shannon index showed that microbiota alpha diversity was greater in the mature milk than that in the colostrum of farm A (Wilcoxon; *p* = 0.84), farm B (Wilcoxon; *p* = 0.84), and farm C (Wilcoxon; *p* = 0.56), indicating the richness and diversity of the milk microbiota (Figure 1A–C). However, the Simpson index of bacterial diversity of farm C indicated that there was no considerable difference between the two lactation stages (Figure S1C). The bacterial diversity was markedly higher in the mature milk than that in the colostrum from farm A and farm B (Figure S1A,B). However, the milk microbiota alpha diversity was not statistically significant in colostrum or in mature milk samples (*p* > 0.05).

**Figure 2:**
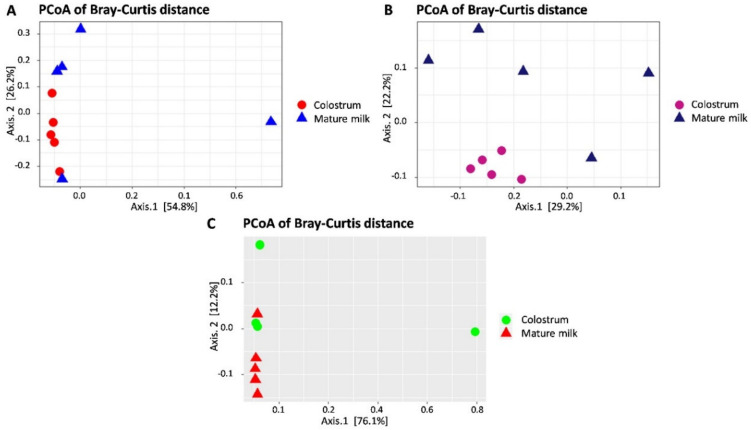
The beta diversity (between microbial diversity) of the colostrum and milk microbiota from three farms, based on principal coordinate analysis (PCoA) plots using the Bray–Curtis distances. Each point on the PCoA plots represents the microbial community from a single sample. Samples with the most similar microbial communities cluster together. The first coordinate (Axis 1) explained 54.8% (**A**), 29.2% (**B**), and 76.1% (**C**) and the second coordinate (Axis 2) explained 26.2% (**A**), 22.2% (**B**), and 12.2% (**C**) of the variation between colostrum and mature milk samples. Colostrum clusters very closely with no discernible difference in farm A and farm B. However, mature milk clusters are long distances from each other (the colostrum and mature milk did not statistically differ).

Comparing the community of bacterial composition in the colostrum and mature milk, the beta diversity analysis was represented by principal coordinate analysis (PCoA). The samples were moderately clustered in accordance with the colostrum and mature milk lactation stages in the PCoA plot of the three different farms (Figure 2A–C). According to the association of OTUs, similar OTUs were classified in the table to estimate the Bray–Curtis distance. Based on the *t*-test and Wilcoxon signed-rank test, we observed clear differences in microbial beta diversity in the colostrum compared with the mature milk of goats.

### 3.2. Total Bacterial Numbers in Dairy Goat Colostrum and Mature Milk from the Different Farms

**Figure 3:**
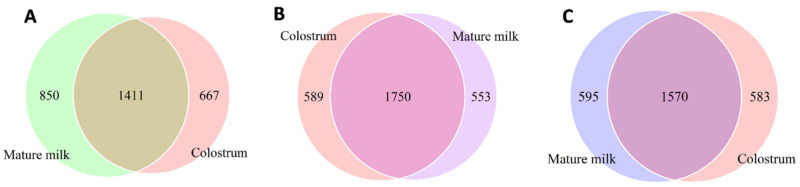
Venn diagram representing the number of bacterial operational taxonomic units (OTUs) in Saanen dairy goat colostrum and mature milk from three (**A**–**C**) farms.

The results of the most prevalent OTUs were used to create Venn diagrams showing the numbers of microbes and variances of each colostrum and mature milk sample (Figure S2) from the different farms (Figure 3A–C). The number of mutual OTUs that were shared by colostrum and mature milk samples from farm A was 1411, representing 48.1% of all OTUs; from farm B the number was 1750, representing 60.5% of all OTUs, and from farm C it was 1570, representing 57.1% of all OTUs. 

### 3.3. Taxonomic Compositions and Differences of Microbiota in Colostrum and Mature Milk of Dairy Goats

**Figure 4:**
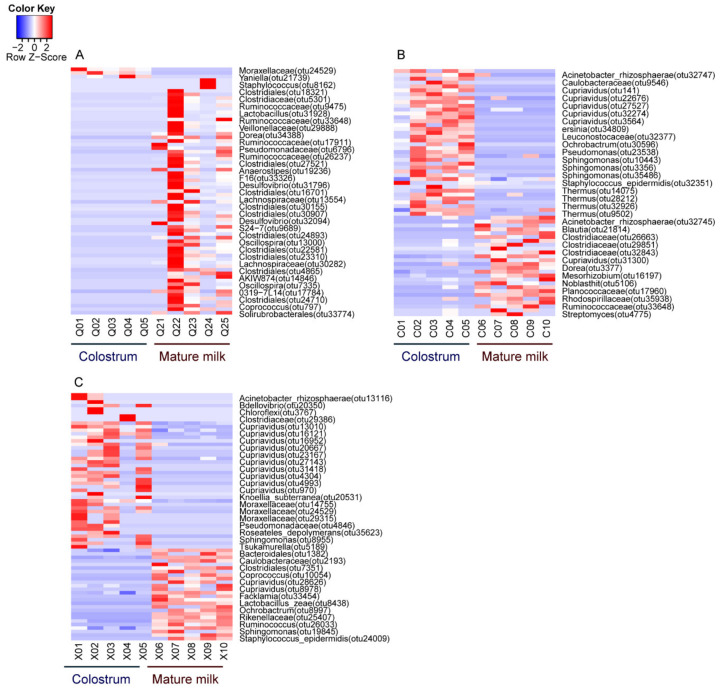
The relative abundance of the top genera between colostrum and mature milk from three farms. The genera with relatively high values are represented in red (0–2) and low values in blue (0–(−2)). Goat milk microbiota composition at genus level was significantly different between the colostrum and mature milk in three farms (**A**–**C**).

In this study, we focused on taxonomic compositions and taxonomic differences of the relatively abundant bacteria obtained from the 16S rRNA gene sequences from colostrum and mature milk samples. Figure 4 displays the average prevalence of the most predominant bacterial genera in the two lactation stages of dairy goat milk from the three different farms.

**Figure 5:**
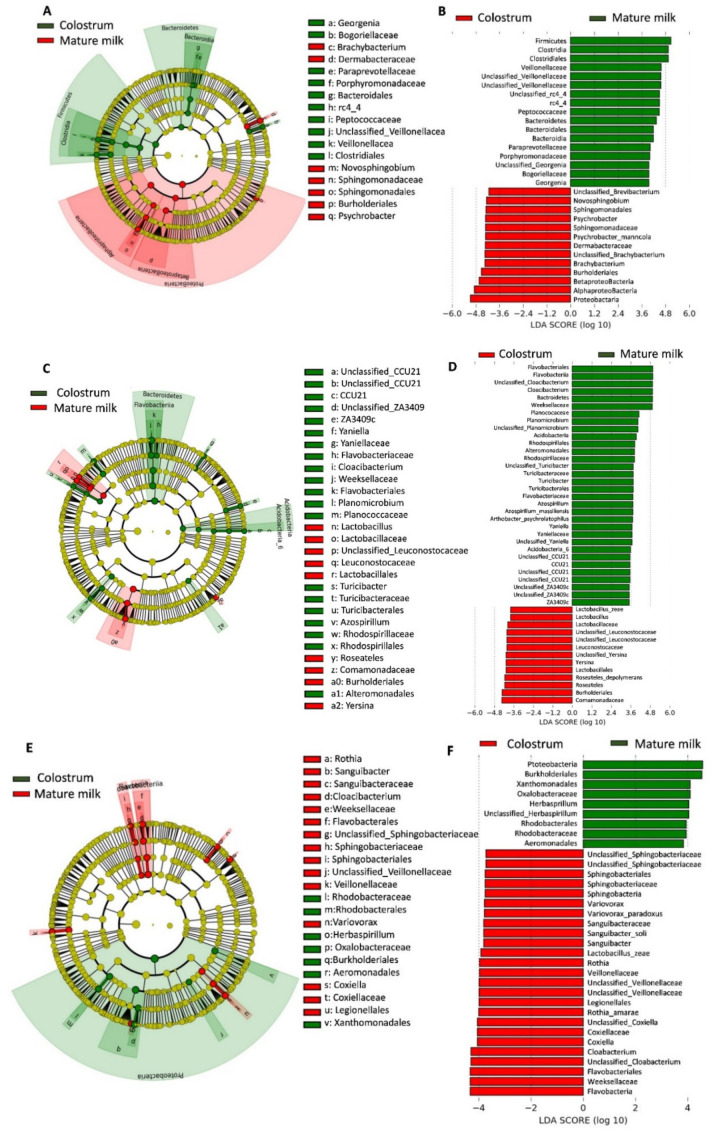
Linear discriminant analysis effect size (LEfSe) analysis of dairy goat colostrum and mature milk microbiota taxonomic differences from three different farms. Differently abundant taxa were identified using linear discriminant analysis (LDA) combined with effect size (LEfSe) algorithm. Histograms of linear discriminant analysis scores of 16S gene sequences in (**A**,**C**,**E**) are shown, with a cut off value of LDA score > 2 (log10). In (**A**,**C**,**E**), colostrum-enriched taxa are indicated with a negative LDA score (red), and taxa enriched in the mature milk are characterized by a positive score (green). (**B**,**E**,**F**) are clipped from LEfSe analysis of differential colostrum and mature milk microbial taxa. The root of the bacterial tree is as the central point and is enlarged into rings for different taxonomic levels, from phylum to genus. The small circles on the rings represent the relative abundance (non-significance in yellow and biomarkers in red and green) of each classification. *Proteobacteria* were significantly enriched in the farm A’s goat colostrum (**B**) and in the mature milk microbiota of farm C (**F**). The relative abundance of Bacteroidetes was higher in the mature milk in the cladogram of farm C (**C**,**D**). *Alphaproteobacteria*, *Betaproteobacteria*, and *Gammaproteobacteria* were significantly abundant classes in the colostrum of farm A.

Furthermore, the abundant microbial taxa were assigned by linear discriminant analysis effect size (LEfSe) with a log LDA (Linear discriminant analysis) score >2.0 and Kruskal–Wallis test and Wilcoxon test (*p* < 0.05) that were statistically different between colostrum and mature milk. LEfSe analysis indicated the taxonomic differences between the colostrum and mature milk in farm A (13; 17 phylotypes) (Figure 5A) and in farm B (13; 32 phylotypes) (Figure 5C). The LDA scores revealed 25 phylotypes with enriched functions of the colostrum microbiota on the negative scale and nine phylotypes of discriminative functions of the mature milk microbiota on the positive scale in farm C (Figure 5E). The relative abundances of *Firmicutes* and *Bacteroidetes* were relatively greater in the mature milk (Figure 5B). We illustrated that *Proteobacteria* were significantly enriched in the colostrum from Farm A (Figure 5B), and in the mature milk from farm C (Figure 5F) which was a potential biomarker for farm C’s goat milk microbiota. The relative abundance of *Bacteroidetes* was higher in the mature milk, while the relative abundances of *Burkholderiales* and *Lactobacillaes* were higher in the colostrum in the cladogram of farm B (Figure 5C,D). The phylum *Proteobacteria*, including the *Alphaproteobacteria*, *Betaproteobacteria*, and *Gammaproteobacteria*, significantly influenced bacterial abundance in the colostrum from farm A (Figure 5B).

A total of twenty-four genera of bacteria dominated in the milk samples from the three different farms (Figure 5B–D). For instance, the predominant bacterial genera in the colostrum of farm A were *Novosphingobium*, *Brachybacterium*, and *Psychrobacter*, while those of the colostrum of farm B were *Lactobacillus*, *Yersinia*, and *Roseateles* and those of the colostrum of farm C were *Rothia*, *Sanguibacter*, *Cloacibacterium*, *Variovorax*, *Sphingobacterium*, and *Coxiella*.

Moreover, predominate bacterial genera detected from mature milk of farm A were *Georgenia*, *Peptostreptococcus*, and *Bacteroidales*. In comparison, those for farm B’s mature milk were *Yaniella*, *Planomicrobium*, *Cloacibacterium*, *Azospirillum*, *Turicibacter*, and *Cupriavidus*, and those from farm C were *Herbaspirillum*, *Rhodobacteraceae*, *Aeromonadales* (Figure 5A,C,E). In addition, relative abundances in individual milk samples of colostrum and mature milk from dairy goats identified by LEfSe were significantly different (*p* < 0.05).

### 3.4. Functional Characterization of Goat Milk Microbiome between Colostrum and Mature Milk

In order to determine the essential role of microbiota in goat colostrum and mature milk, the Phylogenetic Investigation of Communities by Reconstruction of Unobserved States (PICRUSt) [32] program was used to predict metabolic functions from bacterial 16S rRNA genes. The metabolic pathways were classified into six categories by the analysis of the Kyoto Encyclopedia of Genes and Genomes pathway (KEGG, http://www.genome.jp/kegg/pathway.html) database: “Metabolism”, “Genetic information processing”, “Environmental information processing”, “Cellular processes”, “Organismal systems and human diseases”, and “Metabolic pathway”.

**Figure 6:**
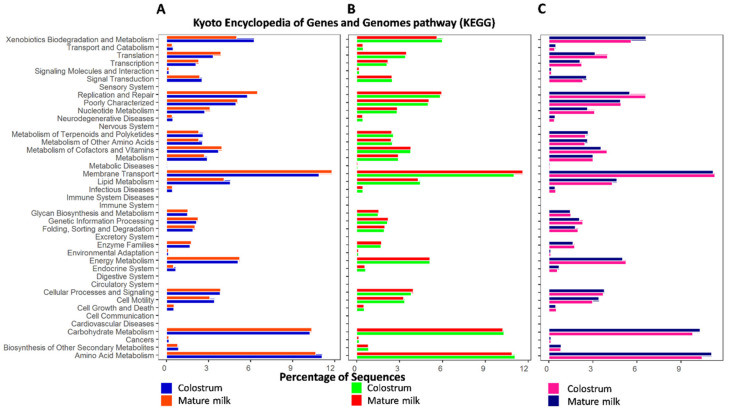
The main categories of the functional analysis based on the Kyoto Encyclopedia of Genes and Genomes (KEGG) pathways. Relative number of genes in microbiota from goat milk (colostrum and mature milk) from three farms (**A**–**C**). Signal transduction (*p* = 0.03), membrane transport (*p* = 0.02), transcription (*p* = 0.02), and amino acid metabolism (*p* = 0.03) pathways were most significantly different in farm A. Carbohydrate metabolism (*p* = 0.0007) was significantly higher in farm B (**B**) goat milk microbiota.

Significantly enriched pathways of milk from farm A include membrane transport (*p* = 0.02) and signal transduction (*p* = 0.03) that are associated with environmental information processing, transport, and catabolism pathways (*p* = 0.05), replication and repair pathways (*p* = 0.05), transcription (*p* = 0.02) and translation (*p* = 0.05) pathways, enzyme families (*p* = 0.04), metabolism of terpenoids and polyketides (*p* = 0.03), nucleotide metabolism (*p* = 0.05), xenobiotics biodegradation and metabolism (*p* = 0.04) and amino acid metabolism (*p* = 0.03) pathways, and excretory system and endocrine system (*p* = 0.04) pathways (Figure 6A). However, levels of environmental information processing-related pathways including membrane transport (*p* = 0.02), transport and catabolism (*p* = 0.04) associated with cellular processes, cellular processes and signaling (*p* = 0.05) and amino acid metabolism (*p* = 0.04) from metabolic pathways, and carbohydrate metabolism (*p* = 0.0007) from organismal systems were significantly higher in milk from farm B (Figure 6B). On the other hand, genes involved in carbohydrate metabolism (9.8%), membrane transport (11.3%), and amino acid metabolism (10.4%) were predominate in milk from farm C (Figure 6C), and there were no statistically significant differences between the two lactation stages of milk samples from farm C.

Ultimately, we found a high prevalence of microbial community and diversity in both populations of colostrum and mature milk from the three different farms (Figure 6).

## 4. Discussion

This study showed 16S rRNA sequencing techniques on V3 and V4 regions of bacterial communities in colostrum and mature goat milk via high-throughput sequencing. Various bacterial species in milk are hypothetically essential for maternal and infant health [33,34]. In the last decade, maternal milk has been shown to harbor a complex bacterial community that helps the establishment of the intestinal microbiota in both newborns and infants [35]. To the best of our knowledge, few studies have reported the microbial composition of goat milk during different lactation stages. The results here indicate that the lactation stage has a significant influence on goat milk microbial and functional composition and bacterial composition was different in colostrum and mature milk. Microbial communities which were found in the colostrum and mature milk could potentially establish goat kid gut microbiota and influence development by their microbial components that have potential for goat health promotion.

In this study, it was shown that bacterial and functional compositions were highly different in all the colostrum and mature milk samples from farms A, B, and C, indicating that lactation stages play crucial roles in the composition of the goat milk microbiota. A previous finding showed that the predominant phyla (*Proteobacteria*, *Firmicutes*, *Acidobacteria*, *Actinobacteria*, and *Bacteroidetes*) from goat milk were similar to the results of the present study (Figure 4A–C). Besides, Moossavi et al. [36] found that *Proteobacteria* and *Firmicutes* were the predominant phyla in human breast milk at 3–4 months postpartum. Our findings elucidated that different lactation stages significantly affect the predominant phyla and genera of milk microbiota. Interestingly, *Proteobacteria* is the largest phylum in colostrum and in mature milk, while *Actinobacteria* is the second largest phylum and were mostly present in colostrum, followed by *Firmicutes* as the third largest phylum in milk. *Firmicutes*, *Proteobacteria*, and *Actinobacteria* are also important phyla in cow milk microbiota, but the specific role of bacteria in the milk microbial community is yet to be determined [37]. Moreover, the phylum *Bacteroidetes* increased in a lactation-dependent manner in both colostrum and mature milk. Therefore, these phyla might play more critical roles in the microbial ecosystem of goat colostrum and mature milk than the other phyla. On the other hand, Zhao et al. [38] identified that *Proteobacteria*, *Firmicutes*, *Deinococcus-Thermus*, *Bacteroidetes*, and *Actinobacteria* were the predominant phyla of camel milk. These results suggested that fresh milk contains high bacterial diversity and is a valuable natural source of various kinds of bacteria. 

Sonnenburg and Bäckhed [39] reported that the gut commensal microbiota contributes to the establishment and the maintenance of innate immune function. In line with that study, we found that the colostrum bacteria are composed of several key components, including *Novosphingobium*, *Brachybacterium*, *Psychrobacter*, *Lactobacillus*, *Yersinia*, *Roseateles*, *Rothia*, *Sanguibacter*, *Cloacibacterium*, *Variovorax*, and *Sphingobacterium*. Moreover, we also identified bacterial genera from mature milk such as *Georgenia*, *Peptostreptococcus*, *Bacteroidales*, *Yaniella*, *Planomicrobium*, *Cloacibacterium*, *Azospirillum*, *Turicibacter*, *Cupriavidus*, *Herbaspirillum*, *Rhodobacteraceae*, and *Aeromonadales* from goat milk samples from three different farms. Other studies also focused on the effect of lactation stage on the immune and physical characteristics of goat colostrum until 5 days postpartum [40].

Several factors, such as the lactation stage, genetic specificity, feedings, geographic locations, and milking equipment, as well as milk transportation and storage, could impact the overall milk microbial community [29]. Doyle et al. [41] studied cow milk lactation stage effects on microbial composition and they found that *Bacteroides*, *Faecalibacterium*, *Campylobacter*, and *Rhodanobacter* to be predominant genera in the mid-lactation stage, which was unlike our study with dairy goat milk. However, the predominant phylum in these studies was *Actinobacteria* in late-lactation milk, which was in resemblance to our investigated mature milk samples from farm A. The lactation stages have been described as an influencing factor on the milk microbiota community [42], as a higher microbial diversity has been mainly reported in mature milk than in colostrum samples.

Tormo et al. [43] described that *Staphylococcus*, *Arthrobacter*, and *Serratia* were the predominant genera in goats of the Languedoc Roussillon and Midi-Pyrenees regions in France. Besides, Zhang et al. [44] found *Enterococcus* and *Lactobacillus* as predominant lactic acid bacteria genera in Saanen and Guanzhong dairy goats. On the other hand, the report of Oliszewski et al. [45] described how Argentinean goat milk and cheeses contain 60% of *Lactobacillus*, showing a strong similarity to farm B goat colostrum of this study. Additionally, *Lactobacillus* isolated from breast milk showed inhibition of adhesion and growth of gastrointestinal pathogens, such as *Escherichia coli*, *Shigella* spp., *Pseudomonas* spp., and *Salmonella* spp. strains [46,47].

Moreover, we analyzed the abundances of the functional metabolism genes of the milk microbiota of goats from the three farms, such as an environmental information processing (*p* < 0.03), cellular processes pathway (*p* < 0.05), genetic information processing pathway (*p* < 0.05), organismal systems pathway (*p* < 0.05), metabolism pathway (*p* < 0.05), and the microbiota functional metabolism gene abundances were significantly different between the colostrum and mature milk. Among these metabolism pathways, carbohydrate metabolism (*p* = 0.0007) presented the highest significance in the Chuan Tou-Cun farm’s goat milk microbiota in this research, with the functional genes involved in amino acid metabolism and carbohydrate metabolism enriched in the goat milk microbiota representing 11.93% and 11.23%, respectively [44].

Further investigation is needed on the effect of goat colostrum and mature milk on the gastrointestinal colonization and the stimulation of the kid immune system. Studies are also needed to identify additional factors that influence the bacterial community; characterize other non-bacterial fractions of the goat milk microbiota, including fungi and viruses; and determine the relationship between the mother’s gut health and milk microbiota composition during different lactation stages. Finally, the effect of milk microbiota during different lactation stages on goat kid gut microbial ecosystems could increase our understanding of the role of the gut microbiota in health and development.

## 5. Conclusions

In conclusion, this research studied the overall compositions of the bacterial communities and diversities of the goat colostrum and mature milk collected from different farms (farms A, B, and C) of dairy goats in China based on 16S rRNA gene sequencing of V3 and V4 regions by using high-throughput sequencing. Additionally, *Proteobacteria*, *Firmicutes*, *Actinobacteria*, and *Bacteroidetes* were the most predominant bacterial phyla in the goat milk from the three different farms of dairy goats. Moreover, the dominance of the microbial communities and their specific compositions in the colostrum and mature milk were diversified, along with changes of the lactation stage period and dominant bacterial genera of the colostrum (*Novosphingobium*, *Brachybacterium*, *Psychrobacter*, *Lactobacillus*, *Yersinia*, *Roseateles*, *Rothia*, *Sanguibacter*, *Cloacibacterium*, *Variovorax*, *Sphingobacterium*, *Coxiella*) and mature milk (*Georgenia*, *Peptostreptococcus*, *Bacteroidales*, *Yaniella*, *Planomicrobium*, *Cloacibacterium*, *Azospirillum*, *Turicibacter*, *Cupriavidus*, *Herbaspirillum*, *Rhodobacteraceae*, *Aeromonadales*) were identified. Therefore, our findings revealed that the goat lactation stages significantly affect the milk microbial community and diversity. Furthermore, the abundance of the microbiota metabolic functions such as the environmental information processing (*p* < 0.03), cellular processes pathway (*p* < 0.05), genetic information processing pathway (*p* < 0.05), organismal systems pathway (*p* < 0.05), and metabolism pathway (*p* < 0.05) of the goat milk were discriminated by PICRUSt and classified by KEGG pathway, where the abundance of the metabolism pathways of the goat milk microbiota were significantly different between the colostrum and mature milk. Altogether, our study provided a good comprehension of the bacterial ecosystem development and differences in goat milk at colostrum and mature milk lactation stages.

## Figures and Tables

**Table 1:** Information of selected colostrum and mature milk samples.

Area	Saanen Dairy Goat	Samples		Given Names
		Colostrum	Mature Milk	
Qian Yang	10	5	5	farm A
Chuan Tou-Cun	10	5	5	farm B
Yang Ling	10	5	5	farm C
Total	30	15	15	30

## Supplementary Materials

The following are available online at https://www.mdpi.com/2076-2615/10/11/1955/s1, Figure S1: Variations of alpha diversity in goat colostrum and mature milk of farms A, B, and C in Simpson index, Figure S2: Venn flower diagram of bacterial OTUs shared between colostrum and mature milk from farms A, B, and C.

## Abbreviations

Ig: Immunoglobulin; DNA: Deoxyribonucleic acid; CTAB: Cetyl trimethylammonium bromide; rRNA: Ribosomal ribonucleic acid; PCR: Polymerase chain reaction; FLASH: Fast length adjustment of short; FASTQ: Format is a text-based format for storing both a biological sequence and quality scores; QIIME: Quantitative insights into microbial ecology; UCHIME: Undetected chimeras; OTU: Operational taxonomic unit; MUSCLE: Multiple sequence comparison by log-expectation; MSA: Multiple sequence alignment; PCoA: Principal coordinate analysis; LEfSe: Linear discriminant analysis effect size; PICRUSt: Phylogenetic Investigation of Communities by Reconstruction of Unobserved States; LDA: Linear discriminant analysis; KEGG: Kyoto Encyclopedia of Genes and Genomes.
