# Supplementary material for: Compositional and Functional Comparisons of the Microbiota in the Colostrum and Mature Milk of Dairy Goats
Source: Animals (Basel). 2020 Oct 23;10(11):1955. doi: 10.3390/ani10111955 (PMC7690780; doi:10.3390/ani10111955)
Supplement: Supplementary file 1 [file animals-10-01955-s001.pdf]

animals

MDPI

Article

Compositional and Functional Comparisons of the Microbiota in the Colostrum and Mature Milk of Dairy Goats

Zhannur Niyazbekova, Xiao-Ting Yao, Ming-Jie Liu, Nomin Bold, Juan-Zhen Tong, Jian-Jun Chang, Ying Wen, Li Li, Yong Wang, De-Kun Chen and Wen-Tao Ma

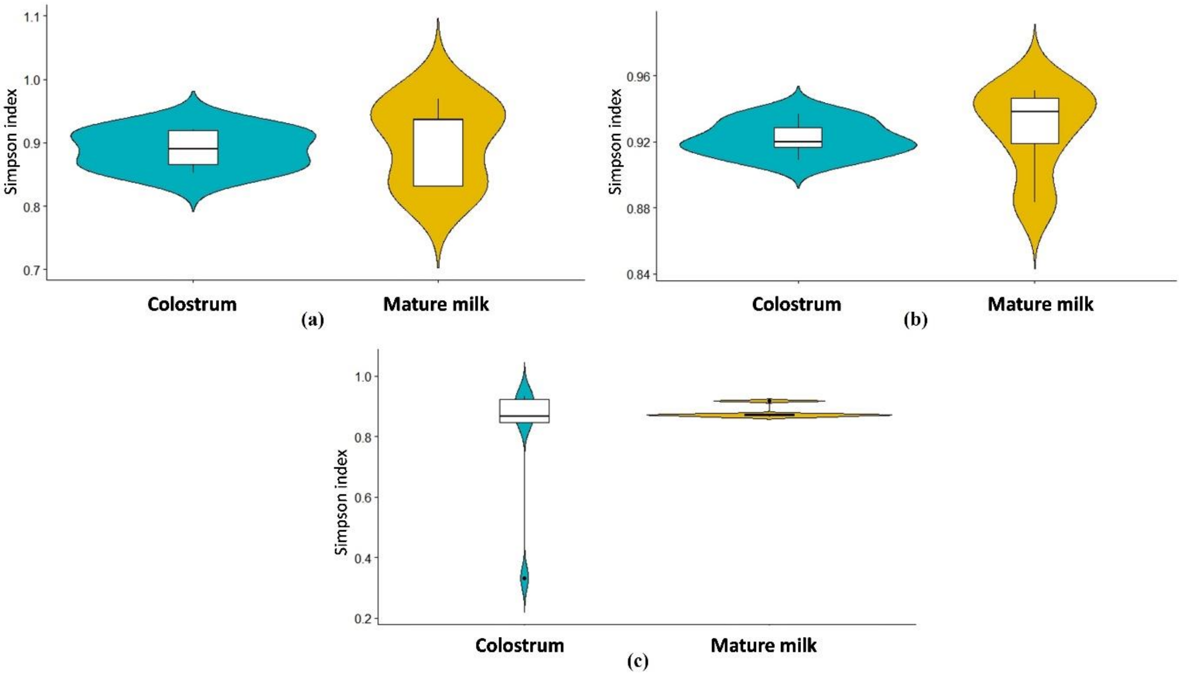

Figure S1. Variations of alpha diversity in goat colostrum and mature milk of A, B and C farms in Simpson index.

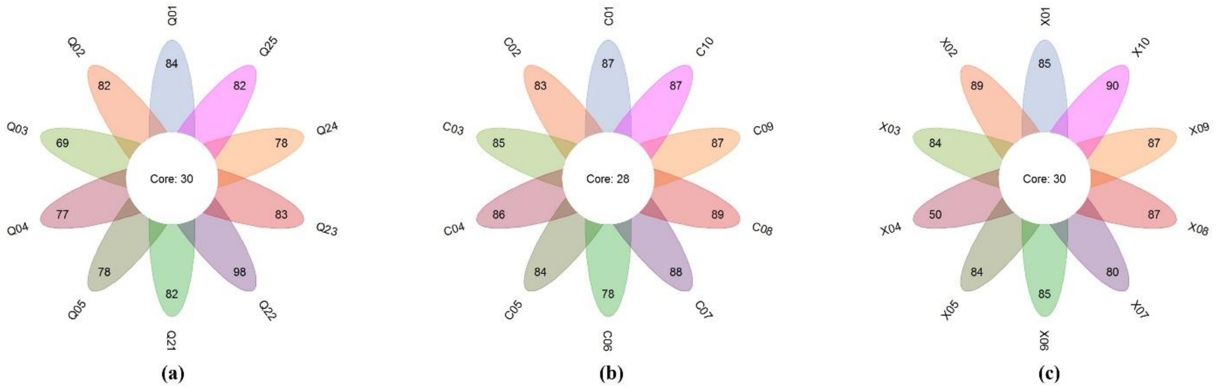

Figure S2. Venn Flower diagram of bacterial OTUs shared colostrum and mature milk from different A, B and C farms. Colostrum samples: Q01, Q02, Q03, Q04, Q05, C01, C02, C03, C04, C05, X01, X02, X03, X04, X05. Mature milk samples: Q21, Q22, Q23, Q24, Q25, C06, C07, X08, X09, X10.

© 2020 by the authors. Licensee MDPI, Basel, Switzerland. This article is an open access article distributed under the terms and conditions of the Creative Commons Attribution (CC BY) license (http://creativecommons.org/licenses/by/4.0/).
